# Digital literacy as a new determinant of health: A scoping review

**DOI:** 10.1371/journal.pdig.0000279

**Published:** 2023-10-12

**Authors:** Maria del Pilar Arias López, Bradley A. Ong, Xavier Borrat Frigola, Ariel L. Fernández, Rachel S. Hicklent, Arianne J. T. Obeles, Aubrey M. Rocimo, Leo A. Celi

**Affiliations:** 1 Intermediate Care Unit. Hospital de Niños Ricardo Gutierrez Buenos Aires, Argentina; 2 Argentine Society of Intensive Care. Management, Quality and Data Committee Buenos Aires, Argentina; 3 Department of Neurology, Neurological Institute, Cleveland Clinic, Cleveland, Ohio, United States of America; 4 Department of Anesthesiology and Intensive Care. Hospital Clínic de Barcelona, Barcelona, Spain; 5 Laboratory for Computational Physiology, Institute for Medical Engineering and Science, Massachusetts Institute of Technology, Boston, Massachusetts United States of America; 6 Research Medical Library, University of Texas MD Anderson Cancer Center, Houston, Texas United States of America; 7 College of Medicine, University of the Philippines Manila Manila, Philippines; 8 Division of Pulmonary, Critical Care and Pain Medicine, Beth Israel Deaconess Medical Center, Boston, Massachusetts, United States of America; University of Manitoba Faculty of Medicine: University of Manitoba Max Rady College of Medicine, CANADA

## Abstract

**Introduction:**

Harnessing new digital technologies can improve access to health care but can also widen the health divide for those with poor digital literacy. This scoping review aims to assess the current situation of low digital health literacy in terms of its definition, reach, impact on health and interventions for its mitigation.

**Methods:**

A comprehensive literature search strategy was composed by a qualified medical librarian. Literature databases [Medline (Ovid), Embase (Ovid), Scopus, and Google Scholar] were queried using appropriate natural language and controlled vocabulary terms along with hand-searching and citation chaining. We focused on recent and highly cited references published in English. Reviews were excluded. This scoping review was conducted following the methodological framework of Arksey and O’Malley.

**Results:**

A total of 268 articles were identified (263 from the initial search and 5 more added from the references of the original papers), 53 of which were finally selected for full text analysis. Digital health literacy is the most frequently used descriptor to refer to the ability to find and use health information with the goal of addressing or solving a health problem using technology. The most utilized tool to assess digital health literacy is the eHealth literacy scale (eHEALS), a self-reported measurement tool that evaluates six core dimensions and is available in various languages. Individuals with higher digital health literacy scores have better self-management and participation in their own medical decisions, mental and psychological state and quality of life. Effective interventions addressing poor digital health literacy included education/training and social support.

**Conclusions:**

Although there is interest in the study and impact of poor digital health literacy, there is still a long way to go to improve measurement tools and find effective interventions to reduce the digital health divide.

## Author summary

This scoping review aimed to investigate the impact of low digital literacy (DL) on health as well as DL and digital health definitions, instruments used to assess it and interventions useful for its mitigation. We performed a comprehensive literature search strategy which identified 53 articles for analysis. Digital health literacy was the most commonly used term to describe the ability to find and use health information using technology. The eHealth literacy scale (eHEALS) was found to be the most utilized tool to assess digital health literacy. Higher scores on the eHEALS were linked to better self-management and participation in medical decisions, improved mental health, and overall quality of life. Overall, this study highlights the need to address the digital health divide and improve access to digital health information and tools for those with low DL. This can be achieved by developing effective interventions to improve digital health literacy, like education/training and social support and increasing awareness of the importance of digital health literacy for better health outcomes. The findings of this study can inform policymakers and healthcare providers on the need to address the issue of low digital health literacy to improve health equity and outcomes.

## Introduction

Digital technologies are transforming health, health care, and public health systems across the world, and they have a great potential to improve population and individual´s health and wellbeing [1]. Technology has advanced in such a way that it has made it possible to expand the type of interactions available between the user and the medical provider or the healthcare systems. Obtaining health information and requesting appointments online, virtual visits, asynchronous digital messaging with healthcare professionals, health tracking wearables and self-monitoring devices, are all technologies available nowadays. These systems bring many advantages; they make it possible to scale information processing, administrative processes and facilitate access to healthcare through virtual visits. Using a video or an online appointment app enables providers to serve hundreds of people that can be attended simultaneously and travel can be avoided for in-person appointments that do not require a physical examination or tests [2]. However, weak governance of digital transformations can also lead to uneven effects globally, increasing health inequities. This reflects the paradox of digital health that we are currently facing: the potential that digital health innovations hold can be transformational for delivering care to underserved population groups (rural areas, aging patients, minorities, or persons with disabilities) but these groups are most likely to be excluded from the digital world through their sociodemographic characteristics [3,4].

The digital transformation, which a priori has many advantages, may contribute to further increasing the inequalities that already exist in access to healthcare thus generating a “digital divide”. “Digital divide” is a term used to encompass a wide range of social differences in access to and use of digital equipment and services, especially personal computers and smartphones, and the ability to access the Internet, both in terms of physical connection and ease of use. As health care becomes more reliant on technology-based tools, the digital divide stands to further exacerbate existing health care access disparities [2].

Digital technologies should be recognized nowadays as a key determinant of health, similar to socioeconomic status, income, education, age, race, ethnicity and gender [4]. Although almost the entire world population now lives within reach of some form of mobile broadband or internet service and mobile phones are becoming ubiquitous, only half of people worldwide use the internet and have basic information and communications technology skills [5]. This gap between internet access and use shows that there are multiple barriers to meaningful access that need to be addressed, especially lack of science, technology, engineering, and mathematics education, digital skills and digital literacy.

Digital literacy can be defined as the varying ability of both children and adults to use digital technologies and understand their risks. It refers not only to the applied technical skills necessary to use and access the internet, but also to the capacity to critically and confidently engage with the online environment. More broadly, as a determinant of health, it has been emphasized that digital literacy substantially interacts with other intermediate health factors and social determinants, to influence both access to digital health resources and wider health equity outcomes [6]. Health literacy–the ability to obtain, read, understand and use health-care information to make appropriate/ informed health decisions [7]–is increasingly becoming a core skill for health-related information on the Internet. Digital health literacy, at first glance, can be regarded as the convergence of digital literacy and health literacy. However, the reality is likely more complex because each competence domain of digital and health literacy may affect one or more competence domains of digital health literacy [3]. On the other side, different terms are used interchangeably in the literature to refer to digital health literacy. According to the National Institutes of Health All of Us Research Program, digital health literacy is “the ability to seek, find, understand, and appraise health information from electronic sources and apply the knowledge gained to addressing or solving a health problem” [8]. Other terms are used depending on the source(s) of the health information. While mHealth literacy focuses on information gathered with the use of mobile devices, eHealth literacy focuses on information gathered from online resources and telehealth literacy specifically focuses on telehealth platforms [1]. In any of these cases, low digital health literacy can carry with it several consequences. Primarily, it can deepen health inequities in an increasingly digitized healthcare landscape. Patients who do not know how to use digital health tools, don’t see the importance of those tools, or can’t access them in their preferred language, ultimately won’t use them. And that puts them at a disadvantage for patient engagement and health improvement. Digital health literacy has recently been acknowledged as one of the “super social determinants of health” because it has implications for the wider social determinants of health [3,6,9] In order to design effective strategies to address this new health determinant, it is necessary to recognise its consequences and the populations it affects. We therefore decided to carry out this scoping review that seek to determine the effects of poor digital literacy on health, specifically looking to define poor digital health literacy, identify populations at risk, the health outcomes affected, its consequences, and interventions targeted to reduce the digital health literacy gap.

## Methods

This scoping review was conducted following the methodological framework of Arksey and O’Malley [10]. The review process was structured according to the following stages:

### Identification of the research question

The research question was stated as, “How does digital health literacy affect health?”. Specific objectives include (1) to define digital health literacy, identify existing assessment tools and groups potentially affected by poor digital health literacy (2) to identify the health outcomes affected by poor digital health literacy and its consequences, and (3) to identify interventions targeted to reduce the digital health literacy gap.

### Identification of relevant studies

On May 11, 2022, a comprehensive search of the literature was constructed and performed by a qualified medical librarian. Medline (Ovid), Embase (Ovid), Scopus, and Google Scholar were queried using both natural language and controlled vocabulary terms for telehealth, digital health, digital literacy, computer proficiency, vulnerable populations, health outcomes, etc. We focused on recent (last 5 years) and highly cited references published in English. Conference abstracts were excluded. The search strategy is detailed in S1 Text. Reference lists of the studies found through database searches were also checked, especially for the systematic reviews and scoping reviews.

### Selection of studies

Original articles on digital literacy and digital health literacy were included if these answered any of the study objectives. Papers that focused on technology use and access instead of digital literacy were excluded. Articles on interventions targeted to reduce the digital literacy gap were excluded if the intervention was not tested in a study population. The initial search returned a large number of results, including non-original articles (systematic reviews and scoping reviews), editorials, letters, commentaries, study protocols, and other publications not deemed relevant that were therefore excluded. Articles without available full text and papers that were not available in English were also excluded. Five authors (MA, BO, AL, XB, AO and AR) independently screened titles and abstracts identified by the electronic search and applied the selection criteria to potentially relevant papers. Any disagreements were resolved by consensus within the group. Data from the selected relevant papers were extracted by one author using a standardised checklist and checked by a second (S1 Template).

### Charting of data

The following key items were obtained based on our consensus as to what information should be collected from the individual studies: author of the article; journal and year the article was published in; article type and objective; setting (country), study population, and sample size; definition and assessment of digital literacy or digital health literacy; health outcomes affected by digital health literacy and its consequences; and interventions targeted to reduce the digital health literacy gap.

The following data were sought for studies that defined digital literacy, digital health literacy, and its related concepts: concept(s) or term(s) used; definition; theoretical framework or model. For studies that employed assessment tools for digital health literacy, the following data were sought: assessment tool; author; elements considered by the assessment tool; aim or intended use of the assessment tool; mode (self-rated versus performance-based); scoring; language; and reliability, Cronbach α. For studies that tackled the correlation between digital health literacy and health outcomes, the following data were sought: health outcomes assessed; and main conclusion on the association between digital health literacy and health outcomes. For studies on interventions targeted to improve digital health literacy, the following data were sought: intervention category; intervention; author; setting; and study population.

### Reporting of results

Pertinent data from the included studies were summarized and analyzed in a narrative, and presented in groups or themes wherever applicable. A critique of the methods and outcomes of the included studies is beyond the scope of this review.

**Fig 1:**
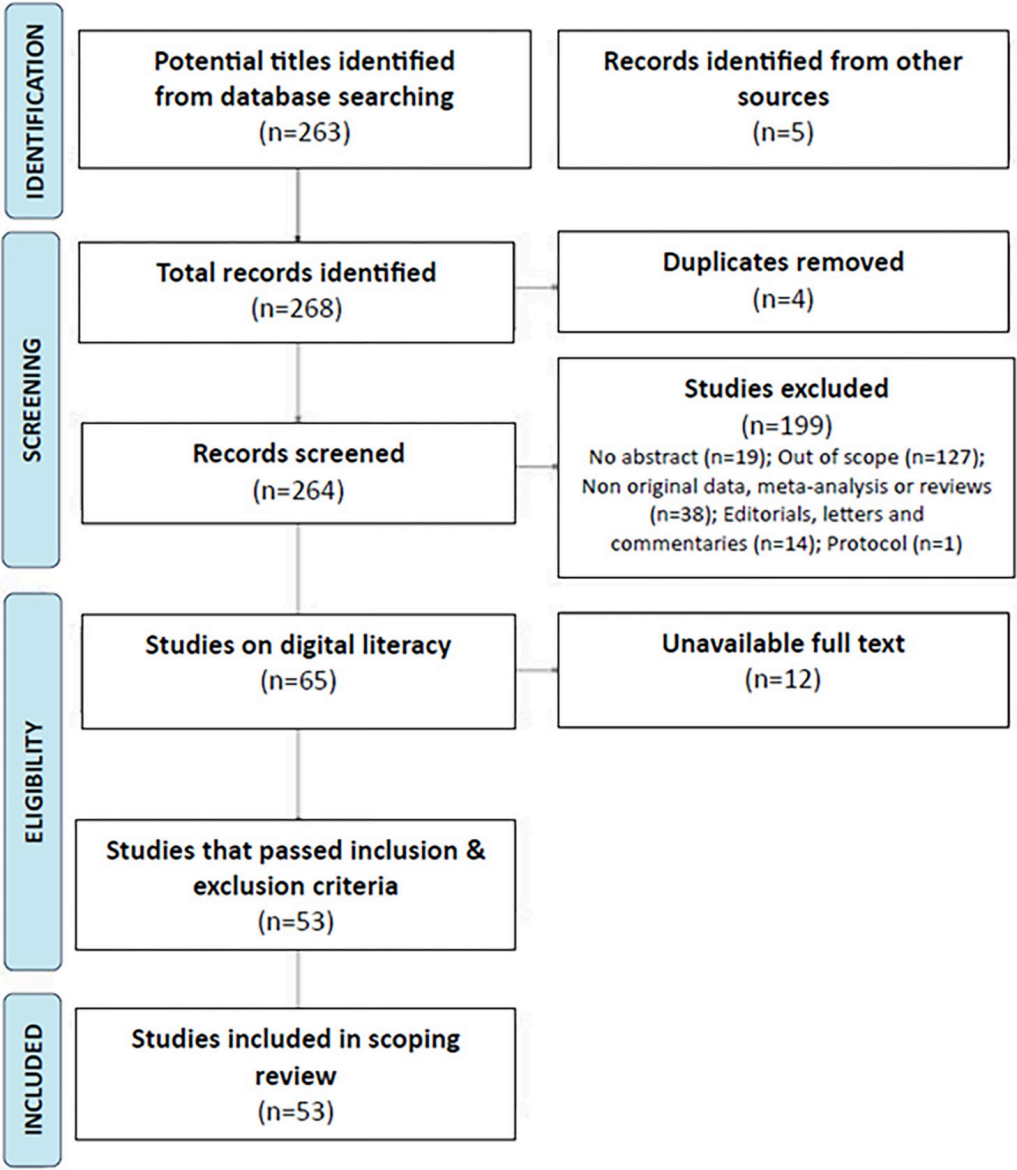
Preferred Reporting Items for Systematic Reviews and Meta-Analyses (PRISMA] flow diagram for the scoping review of digital health literacy.

**Fig 2:**
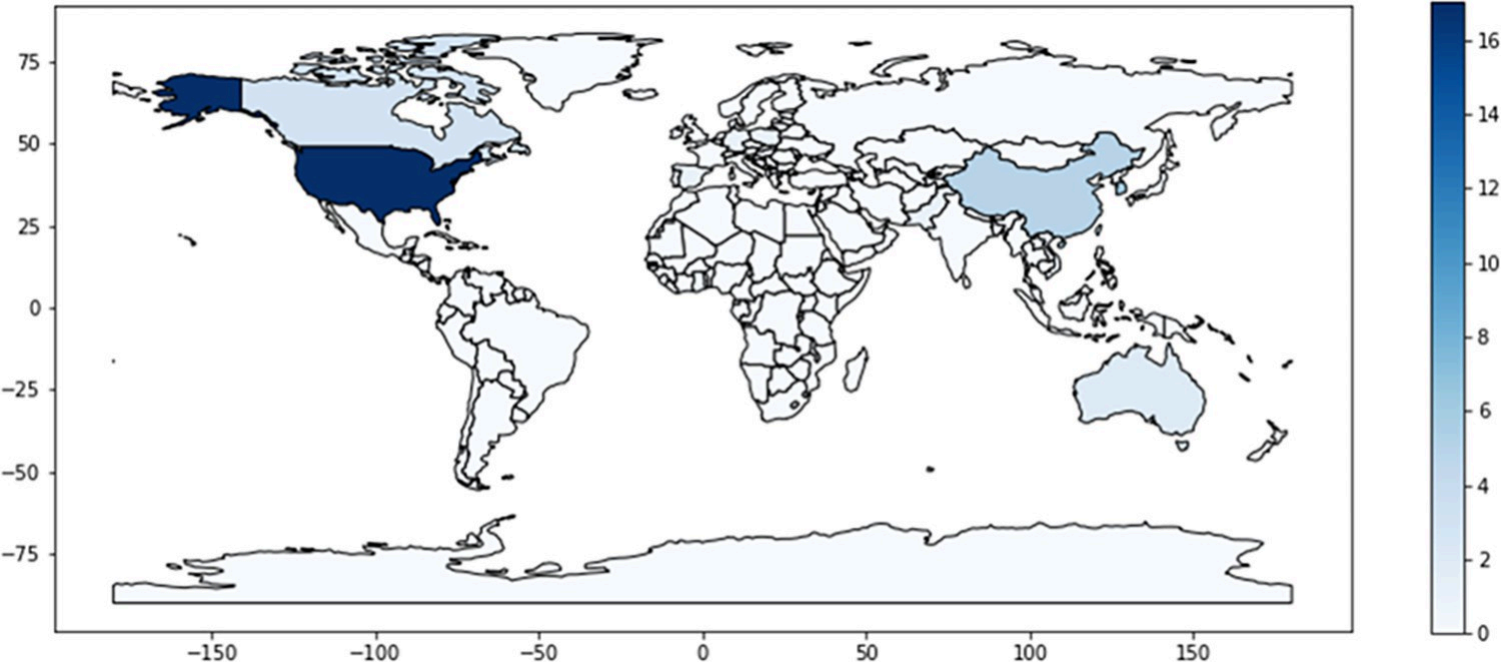
Nationality of the population of origin in the selected articles. This figure shows the geographic gap in relation to the origin of the selected articles. If we consider that the determinants of poor digital health literacy (age, level of education, belonging to an ethnic minority) could be the same globally, this figure is indicative of the recognition and interest of the problem in the different countries. Figure created using the Natural Earth base layer from www.naturalearthdata.com under the PDDL license https://opendatacommons.org/licenses/pddl/.

This review was not registered and neither a protocol was prepared. All data are in the manuscript and/or supporting information files. The data used to construct Figs 1 and 2 are reported in table format in S1 Data.

## Results

A total of 268 articles were identified in the study. Of these, 263 came from the electronic search and five came from the references of the literature and systematic reviews identified from the electronic search. Fig 1 lists the number of studies included and excluded per step according to the diagram adapted from the Preferred Reporting Items for Systematic Reviews and Meta-Analyses: The PRISMA Statement [11].

Four duplicates were identified. Of the remaining articles, 199 did not proceed beyond the screening stage due to any of the following: no abstract; out of scope; non-original data, meta-analysis, or reviews; editorials, letters, or commentaries; or protocol. Twelve articles were further excluded due to unavailable full text.

### Characteristics of included studies

The main characteristics of the included studies are described in S1 Table.

The 53 studies that reached analysis were published in a diverse range of journals. Among the included studies, the oldest was published in 2016 [12] while the most recent ones were published in April 2022 [13, 14]. The majority of the studies were published in 2021 (19/53; 35.85%) followed by 2020 (15/53; 28.30%).

Of the 53 included studies, 44 were cross-sectional studies (44/53; 83.01%). Only six (6/53; 11.32%) studies were longitudinal: three of them experimental [14–16], the rest pre-post observational studies [17–19]. The last three studies were mixed methodology studies [20–22].

Most studies (20/53; 37.74%) were conducted in North America (17 of them in the United States of America, 2 in Canada, and 1 in Mexico), 17 in Asia (6 in China, 6 in Korea, 1 in Pakistan, 1 in Taiwan, 1 in Vietnam, and 1 in multiple countries), 9 in Europe, 2 in Australia and 1 in Africa (Fig 2).

### Definition of digital health literacy

Different terms were used across the studies to refer to digital health literacy, including electronic health literacy, eHealth literacy, mHealth literacy, telehealth literacy, and mobile health proficiency. Among these, the most frequently used in the included studies is eHealth literacy (30/53; 56.60%) followed by digital health literacy (12/53; 22.64%). These concepts similarly refer to the ability to find and use health information with the goal of addressing or solving a health problem using technology. However, these are differentiated by the source(s) of the health information [23–26]. mHealth literacy focuses on information gathered with the use of mobile devices [25] while eHealth literacy focuses on information gathered from online resources [23]. Telehealth literacy specifically focuses on telehealth platforms [27].

In the included studies, digital health literacy was most often used interchangeably with the earlier term eHealth literacy [23]. Whereas eHealth literacy is limited to information from Web 1.0 platforms viewed by users in a passive manner, digital health literacy incorporates information from Web 2.0 platforms with interactive content including social media, blogs, and video sharing sites [23–25]. Digital health literacy is therefore a broader concept compared to eHealth literacy.

A more specific concept related to digital health literacy encountered in one included study is digital healthy diet literacy, defined as the ability to access and appraise digital healthy-diet-related information to improve healthy eating behavior and health outcomes [28].

The term digital literacy instead of digital health literacy was used in seven of the included studies (7/53; 13.21%). Compared to digital health literacy and eHealth literacy, digital literacy is a broader term as it refers to the ability to find and apply digital information. Other terms used by the included studies for the same concept include digital competency [29], digital capability level [30], mobile phone digital literacy [31], new media literacy and technological literacy [32].

The conceptual framework of eHealth literacy by Norman and Skinner [23] was employed by the majority of studies (32/53; 60.37%). Four studies [33–36] employed the e-health literacy framework by Norgaard et al. [24] and one study [37] employed the transactional model of ehealth literacy by Paige et al. [38]. In Norman and Skinner’s framework, eHealth literacy is likened to the pistil that holds the petals of the lily flower together, similar to how eHealth literacy ties together six core skills: traditional literacy, health literacy, information literacy, scientific literacy, media literacy, and computer literacy [23]. In contrast, the framework by Norgaard et al. [24] encompasses domains largely dependent on the individual (domain 1: ability to process information; domain 2: engagement in own health), domains largely dependent on the system (domain 6: access to digital services that work; domain 7: digital services that suit individual needs), and domains on the dynamics between the individual and system (domain 3: ability to actively engage with digital services; domain 4: feel safe and in control; domain 5: motivated to engage with digital services). The transactional model of eHealth literacy, on the other hand, highlights the transactional features central to eHealth literacy and outlines four operational skills: functional, communicative, critical and translational [38].

In S2 Table, we detail the concepts related to digital health literacy and its corresponding theoretical framework employed in the included studies.

### Measurement of digital health literacy

Twenty different assessment tools were employed by the included studies to assess digital literacy and/or digital health literacy (Table 1).

**Table 1:** Assessment tools for digital literacy and digital health literacy.

Assessment Tool	Elements	Mode	Language	Studies which apply the tool
Digital competency questionnaire [29]	[1] Information processing; [2] content creating; [3] communication; [4] safety; [5] problem solving	Self-rated, Likert scale	not specified	[29]
Digital capability level [30]	[1] PC usability; [2] mobile device usability	Self-rated, Likert scale	Korean	[30]
Computer Proficiency Questionnaire [CPQ] [39]	[1] computer basics; [2] printing; [3] communication; [4] Internet; [5] calendar; and [6] entertainment	Self-rated, Likert scale	English	[42]
Digital literacy tool [40]	[1] technical competencies [software usage ability and smart device usage ability], [2] mind competency	Self-rated, Likert scale	Korean	[69]
Digital Literacy Evaluation [DILE] [41]	[1] Use and knowledge of the computer; [2] Use and knowledge of the Internet; and [3] Knowledge of home and daily life devices	Performance-based	Spanish	[19]
Mobile Device Proficiency Questionnaire [MDPQ] [42]	[1] mobile device basics; [2] communication, data and file storage, Internet, calendar, entertainment, privacy, and troubleshooting and software management	Self-rated, Likert scale	English	[42]
eHealth Literacy Scale [eHEALS] [43]	[1] traditional literacy; [2] computer literacy; [3] information literacy; [4] health literacy; [5] media literacy; [6] science literacy	Self-rated, Likert scale	Chinese, English, Japanese, Korean, Vietnamese	[14–16–18,28,35,52,55–61,65,66,70–74]
eHealth Literacy Questionnaire [EHLQ] [44]	[1] using technology to process health information; [2] understanding of health concepts and language; [3] ability to actively engage with digital services; [4] feel safe and in control; [5] motivated to engage with digital services; [6] access to digital services that work; [7] digital services that suit individual needs	Self-rated, Likert scale	Chinese, Danish, English	[21,69]
eHealth Literacy Assessment [eHLA] Toolkit [36]	[1] functional health literacy; [2] self-assessed health literacy; [3] familiarity with health and health care; [4] knowledge of health care; [5] familiarity with technology; [6] technology confidence; [7] incentives for engaging with technology	Self- rated and performance-based	Danish, English	[36]
electronic Health Literacy Scale [e-HLS] [12]	[1] communication; [2] trust; [3] action	Self-rated, Likert scale	English	[12]
Digital Health Literacy Instrument [DHLI] [45]	[1] operational skills; [2] navigation skills; [3] information searching; [4] evaluating reliability; [5] determining relevance; [6] adding content; [7] protecting the privacy	Self- rated and performance-based	Chinese, Danish, English, Korean	[13,22,52–54]
Digital Health Literacy Assessment [DHLA]] [46]	[1] self-assessment of digital health literacy; [2] how convincing people found internet health information from different sources; [3] trust in health information from folklore and customs	Self-rated, Likert scale	Chinese	[46]
Digital Health Literacy Instrument in Relation to COVID-19 Information [COVID-DHL-K] [47]	[1] Searching the web for information on coronavirus; [2] Adding self-generated content on coronavirus; [3] Evaluating the reliability of coronavirus-related information; [4] Determining personal relevance of coronavirus-related information; [5] Protecting privacy on the Internet	Self-rated, Likert scale	Korean	[47]
Digital Health Literacy Assessment Tool [DHLAT] [48]	[1] functional health literacy; [2] health literacy self-assessment; [3] familiarity with health and health care; [4] knowledge of health and disease; [5] technology familiarity; [6] technology confidence; [7] incentives for engaging with technology	Self-administered, open-ended questionnaire	English	[48]
Digital Healthy Diet Literacy [DDL] [49]	ability to [1] find reliable and accurate healthy diet information on the internet, [2] understand healthy diet information and dietary guidelines on the internet, [3] judge whether healthy diet information on the internet is applied for individuals, and [4] apply healthy diet information from the internet into individuals’ daily lives to eat healthily	Self-rated, Likert scale	Vietnamese	[49,55]
Transactional eHealth literacy instrument [TeHLI] [37]	[1] Functional eHealth literacy; [2] Communicative eHealth literacy; [3] Critical eHealth literacy; [4] Translational eHealth literacy	Self-rated, Likert scale	English	[37]
Telehealth Literacy Screening Tool [TLST] [27]	[1] biopsychosocial background including access to technology; [2] technological literacy screening; [3] eHealth literacy screening	Self-rated, Likert scale	English	[27]
Readiness and Enablement Index for Health Technology [READHY] Tool [33]	7 eHealth Literacy Questionnaire [eHLQ] dimensions: [1] using technology to process health information; [2] understanding of health concepts and language; [3] ability to actively engage with digital services; [4] feel safe and in control; [5] motivated to engage with digital services; [6] access to digital services that work; [7] digital services that suit individual needs; 4 Health Education Impact Questionnaire [heiQ] dimensions: [1] self-monitoring and insight; [2] constructive attitudes and approaches; [3] skill and technique acquisition; [4] emotional distress; 2 HLQ dimensions: [1] understanding and support by healthcare providers; [2] social support for health	Self-rated, Likert scale	English	[33]
Everyday Health Information Literacy–10 [EHIL-10] [50]	[1] health information–seeking ability; [2] described health information evaluation ability; [3] health information consciousness; [4] health information application ability	Self-rated, Likert scale	Chinese	[50]
Mobile eHealth Literacy Questionnaire [51]	[1] eHealth literacy [eHL]; [2] mHealth literacy [mHL]; [3] mobile eHealth preference	Self-rated, Likert scale	English	[51]

Forty-five of the included studies (45/53; 84.90%) used at least one of these assessment tools. (S3 Table) The tools differ in terms of evaluation elements, applicable groups, and intended use. Six of these specifically focus on digital literacy [29,30,39–42], six on eHealth literacy [12,33,36,37,43,44], four on digital health literacy [45–48], one on digital healthy diet literacy [49], one on telehealth literacy [27], one on health information literacy [50] and one on a combination of eHealth and mHealth literacy [51]. Most of these tools are self-rated Likert scales except for eHealth Literacy Assessment [eHLA) toolkit [36] and Digital Health Literacy Instrument [DHLI] [45] which both employ a combination of self-rated and performance-based assessment, and Digital Literacy Evaluation [DILE) tool [41], which is performance-based.

The eHealth literacy scale [eHEALS) by Norman and Skinner [43] is the most widely used in the included studies (24/53; 45.28%). Five studies [13,22,52–54] used the DHLI [45], and two studies [49,55] used the Digital Healthy Diet Literacy [DDL) tool [49].

Twelve of the twenty assessment tools identified are available in English, five in Chinese, three in Danish, four in Korean, two in Vietnamese, and two in Spanish. Four of the assessment tools are available in several languages. The included studies used different versions of eHEALS (English [15,18,28,56–60], Spanish [17,61], Chinese [62–64], Korean [52,65,66], and Vietnamese [55,67]), eHealth Literacy Questionnaire (EHLQ) (Chinese [68], English [21], Danish [21]), DHLI (Chinese [53], Danish [22], English, [13,53], Korean [52]), and eHLA toolkit (Danish [36], English [36]).

### Groups affected by lower digital literacy

Several of the included publications report that the level of health digital literacy was associated with gender, age and level of education.

Abdulai et al. [56], performed a survey with 268 respondents aiming to examine the digital literacy of lay consumers of online COVID-19-related information in Ghana. In their study the authors describe that males were more likely than females to have high digital literacy related to internet-based information. At the same time, according to this survey, digital literacy was likely to be lower among older people despite being the group more likely to suffer from COVID-19 complications.

Similarly, Guo et al. [62], in a random cohort of adults in Hong Kong, examined socioeconomic disparities in seeking web-based information on COVID-19 and eHealth literacy, and their associations with personal preventive behaviors during the COVID-19 pandemic. In this study the eHL and mHL literacy scores had significant and negative associations with age [eHL, r = -0.380, P < .001; mHL, r = -0.398, P = .036]. The results also show that the participants with higher education had a greater level of mobile eHealth literacy.

The association of level of education and digital literacy was also described by Adil et al. [28] In the survey that they performed among a sample of university students, the authors report that belonging to different categories of educational attainment affects the level of usage and of expertise in digital health literacy in varying ways. This study concludes that educational level is the major factor for unequal response towards digital health literacy. The study furthermore depicted that the students of BS/Master, MS/MPhil and PhD are substantially different from each other in their level of usage and expertise.

In order to recognize patient´s perspectives of the principal causes of digital divide, Alkureishi et al. [2] conducted 54 semi structured telephone interviews with adult patients and parents of pediatric patients who had virtual visits (phone, video, or both) between March and September 2020 at the University of Chicago Medical Center (UCMC) primary care clinics. The most common subtheme cited by the participants as a cause of medical divide was advanced age, which was considered a major contributor and limitation to their ability to learn and navigate technology. Cognitive and medical impairments, including memory loss and hearing and visual impairments, were also challenges that contributed to the digital divide among older individuals.

At the same time, the study of Aponte et al.[61], which evaluated the Spanish version of the eHEALS with an older Hispanic adult sample in a senior organization of a Spanish neighborhood in New York reported that the highest item in the eHEALS results was related with the importance that the persons assigned to being able to access health resources on the Internet (mean eHEALS: 4.4 (DS 0.7)) while the lowest item was the one related to their ability to use the Internet to answer their questions about health (mean eHEALS: 3.2 (SD: 12)), indicating that respondents knew how to find health-related information on the internet but were not confident in using that information to make health decisions.

In addition, a secondary analysis of the CALSPEAKS survey performed by Berkowsky et al. [70], showed that in the group of respondents older than 65 years of age, level of education (less of high school, high school, some college, associate’s degree, bachelor’s degree or postgraduate degree) and measures of digital experience and skill (e.g frequency of Internet use, breadth of Internet activities performed regularly) had strong and consistent associations with eHealth literacy.

### Digital health literacy and health outcomes affected

Of the 53 studies included, only 13 (24.53%) studies reported on how digital literacy affected health outcomes. Health outcomes reported encompass health promotion, quality of life, mental and psychological states, disease prevalence, and health status. Table 2 describes the health outcomes related to the level of digital health literacy.

**Table 2:** Health outcomes related to digital health literacy.

Health Outcomes and Consequences	Main conclusions	Literature cited
Health promotion	Individuals with better digital health literacy were able to self-manage and engage in their own medical decisions and showed greater ability in following preventive public health measures.	Alkureishi et al. [2], Kim et al. [66], Li et al. [63], Li et al. [64], Perestelo-Perez et al. [74]
Quality of life	Individuals with good digital health literacy had higher quality of life, sense of purpose, and sense of optimism.	Alkureishi et al. [2], Jang et al. [69], Nguyen et al. [55], Papp-Zipernovszky et al. [35]
Disease prevalence	Individuals with better digital health literacy have reported lower numbers of disease cases.	Guo et al. [51], Kemp et al. [34], Perestelo-Perez et al. [74]
Mental and psychological states	Individuals with higher digital health literacy are better equipped to manage their mental state. Individuals are also able to avoid negative emotions and prevent psychological issues.	Leung et al. [53], Yang et al. [68]
Health status	Individuals with higher digital literacy reported better long term disease control.	Guo et al. [51], Kemp et al. [34]

The most frequent health outcome reported was health promotion. Five of 13 studies (38.46%) reported health-promoting behaviors, including health responsibility, stress management, exercise behavior, self-realization, and social support. Both quality of life and disease prevalence were the second most common reported health outcomes in this study (4/13, 30.77%). This was followed by mental and psychological states (2/13; 13.58%), which involves managing negative emotions, meta-cognition, and psychological well-being. Lastly, 2/13 (15.38%) studies assessed how digital literacy affected the health status of patients. Individuals with better digital literacy were likely to have better disease control (type 2 diabetes patients) [51].

According to the patient’s perspectives, digital literacy can limit access to online patient portals. Without access to these tools, less technologically able individuals experience challenges in care coordination such as scheduling visits, communicating with their clinicians, and facilitating referrals and tests. Patients also consider that the divide can worsen personal health care outcomes because of limited opportunities and resources to coordinate health care needs. Less technology- savvy individuals had significant challenges to access to COVID-19 vaccine and to use online scheduling portals [2].

### Interventions that address poor digital health literacy

Of the included studies, only 9 (9/53; 16.98%) evaluated interventions addressing poor digital health literacy (Table 3). These interventions can be divided into 2 categories: education and training, and social support. Majority of the interventions which significantly improved digital health literacy were under education and training. Under education and training, massive open online courses, university training in e-health through tutoring, and online video-based portal training were reported to improve digital health literacy of both children and adults. On the other hand, 2 out of 9 reported interventions were categorized under social support. Social support from technology-savvy family members, professionals, and peers were shown to improve digital health literacy of adults and older adults.

**Table 3:** Interventions that address poor digital health literacy.

Author	Intervention Category	Setting	Study population	Intervention	Result of Intervention
Alvarez-Perez et al. [20]	Education and Training	Italy, Spain, Sweden	Adults and adolescents with type 1 and type 2 diabetes	Cocreation of massive open online courses [MOOCs], “a type of open educational resource used to improve education and practice, easily applicable to empower patients with chronic conditions to find quality, equitable, patient-centered education aimed at better health outcomes”	“In the 3 subsamples in which self-perceived digital health literacy was assessed [Italy, Spain, Sweden], significant pre-post improvements [via eHEALS] were observed in the appraising information scale and at least 1 out of the other 2 dimensions [ie, finding and understanding].”
Perestelo-Perez L, et al. [74]	Education and Training	Spain, Italy, Belgium, the United Kingdom, Sweden, Denmark and Estonia	[a] children; [b]adolescents; [c] pregnant and lactating women; [d] people over 60 years of age; and [e] people with type 1 and type 2 diabetes	Co-design a series of MOOCs	“70–80% of the participants showed an excellent integration of Digital Health Literacy [DHL] competencies by obtaining all the correct answers, after having completed the MOOCs.” “Higher scores on the eHeaLS Scale in DHL were observed in all cohorts after the use of the MOOCs.”
De la Hoz et al. [17]	Education and Training	Spain	Bachelor degree students	“University training in eHealth through interventions based on a cooperative active methodology [tutoring]. . .”	“The results at the end of the intervention clearly show that university training in eHealth through interventions based on a cooperative active methodology [tutoring] is an effective means of improving searches for information and use of digital resources relating to health science knowledge.”
Hyman et al. [18]	Education and Training	Canada	Children aged 9–14 years [Intermediate elementary students Grade 4–7]	Learning for Life [L4L], a school-based intervention for increasing digital health literacy [DHL] and healthy lifestyle behaviors in children aged 9–14 years	“From pre- to post-intervention, students’ digital health literacy increased [p = 0.009], but decreased from post-intervention to 2-month follow-up [p < 0.001].”
St. Jean et al. [48]	Education and Training		middle school students [ages 12–15]	After-school program [HackHealth]	“HackHealth participants tended to understand what search engines are and were generally able to formulate appropriate queries; on the other, they were largely unaware of how search engines actually work and were not familiar with trustworthy websites to which they might directly navigate when they need credible health-related information”
Lyles et al. [15]	Education and Training	USA	English-speaking adults diagnosed with chronic conditions [hypertension, depression, diabetes, anxiety, asthma or copd, heart disease, heart failure, chronic kidney disease]	Online video-based portal training	“An online video-based portal training resulted in a significant increase in the eHealth literacy scale over time [14.4 to 16.2, p<0.001]. Participants’ self-reported confidence in using the website and eHealth digital literacy appeared to be the most malleable to improvement post-training.”
Martínez-Alcalá et al. [19]	Education and Training	Spain	Older adults	Digital Literacy Workshops	“In all cases, pre vs post scores of the DILE [Digital Information Literacy Evaluation] were significant”
Abdulai et al. [56]	Social support	Ghana	Adults and older adults	Intergenerational help from technology-savvy family members such as children and grandchildren	“Many patients, particularly older adults, reported having a greater dependency on tech-savvy younger relatives and friends, highlighting the importance of intergenerational assistance to help less technology literate patients navigate technology”
Tsai et al. [16]	Social support	USA	Older adults aged 69 to 91 years old	Support from three areas: family, professionals, and peers.	Primary source of help in the learning process about digital health: “71% [n = 15] talked about support from family, 19% [n = 4] went to professionals such as the Apple store, and 10% [n = 2] had support from peers”

## Discussion

Digital determinants of health, like insufficient technology access and digital literacy, are currently recognized as Social Determinants of Health (SDOH). However, they should not be considered merely the sixth domain on the list of determinants of health, as they are major controllers of every SDOH and the environment in which they can be accessed fully.

Instead, digital determinants should be considered “super determinants of health” taking into account that they are drivers of each SDOH profoundly influencing whether they are functional or dysfunctional and potentially impacting one’s overall health and quality of life.

Decision makers, health professionals, and researchers must consider and address the effects of digital determinants on population health in order to design and implement improvement strategies. Therefore, we decided to perform this scoping review with the aim to summarize current knowledge about digital literacy and its consequences on health, specifically searching to identify most frequently impacted groups, health outcomes affected and proposed interventions targeted to reduce the so-called “Digital divide”.

The body of existing literature on the topic is vast and growing, especially in the last two years. Although it is rich in definitions and author´s proposals of strategies to improve digital health literacy, publications that describe specific health outcomes affected or proven interventions are scarce. The overall level of evidence of the analyzed studies was low; only 4 of the 53 articles included in the review have levels of evidence of II and III corresponding to small randomized controlled trials and case control studies respectively; the rest are level V corresponding to observational studies and case series [75]. Most of the samples of the revised publications were small sized.

Although most studies show that patients with lower levels of digital literacy and access to technology are more likely to belong to marginalized backgrounds, including older persons, black and hispanic populations and non-English speaking patients, the vast majority of the studies were conducted in North America, Europe and China. In the selected papers, Africa, South America and a large part of Asia and Oceania are underrepresented and surely coincide with the regions most affected by the digital health literacy gap, since in these areas a large part of the population has similarities at the socioeconomic and educational level with the groups we have described as most affected by the digital health gap.

Defining and measuring digital health literacy should be the first step for bridging the digital divide. However, the definitions used in the different studies are heterogeneous as well as the instruments used for its measurement. Digital literacy can be considered an umbrella term for many different technologies (internet, mobiles, social media, etc) and affects various areas of human lives such as education, business, health, governance among others. It has been defined as “the skills required to achieve digital competence, the confident and critical use of information and communication technology for work, leisure and communication” [26]. At the same time, health literacy can be defined as “the degree to which individuals can obtain, process, understand, and communicate about health-related information needed to make informed health decisions” [23]. At first glance “Digital Health literacy” can be regarded as the convergence of digital literacy and health literacy [76]. However, the reality is more complex. In most published studies, both health and digital literacy are conceptualized through competency-based frameworks. Health literacy is elaborately expressed through a matrix of four dimensions (access/obtain information relevant to health, understand information relevant to health, process/appraise information relevant to health, and apply/use information relevant to health) that are applied across three domains (healthcare, disease prevention, and health promotion) [8]. A European Commission framework on digital competencies takes a similar approach to digital literacy by depicting five dimensions [information and data literacy, communication and collaboration, digital content creation, safety, and problem- solving], each with four to six sub-dimensions that illustrate a core competence of digital literacy [77]. The relationship between digital, health, and digital health literacy is a multi-dimensional one where each competence domain of digital and health literacy may affect one or more competence domains of digital health literacy [78], but certain competencies of digital health literacy may not be covered by neither digital literacy nor health literacy [7]. As an example of this statement, Abdulai reported that educational status, frequency of using the internet, and using the internet for social media and entertainment purposes were not significant predictors of digital literacy related to online COVID-19 information [56]. Though the overall literacy level was high, respondents had a relatively lower mean score on questions that indicate they may have some challenges locating the right kind of COVID-19 online resources, as well as a limited ability to distinguish high-quality information from those reflecting personal opinions or anecdotal stories [56]. Likewise, Guo et al report in their survey performed in a sample of people with diabetes in 3 taiwanise hospitals that, although they were confident in using mobile eHealth and technology, only 1.6% used health apps or adopted these tools in their daily lives [51].

The complexity and multidimensionality of health and digital literacy highlight the need to conceptualize digital health literacy in the context of a competence framework.

The analysis of the instruments used for digital health measurement showed that eHealth Literacy Scale (eHEALS) was the most widely used in several countries and populations.

This is an 8-item scale (measured on a scale of 1 = strongly disagree to 5 = strongly agree) for measuring participants’ self-reported skills at finding, appraising, and using health related information available on the internet. Higher scores represent higher perceived digital literacy, while lower scores indicate lower perceived literacy. The eHEALS has demonstrated considerable reliability and validity in studies performed in various settings (countries with different profiles of resources) and social groups (college students, undergraduate nurses, older adults). It was translated in different languages and was also adapted by some authors, limiting it to specific resources as COVID-19 resources instead of general health resources as contained in the original instrument [56] or particular topics such as digital dietary literacy [55]. Although eHEALS is frequently used, it is increasingly recognized that the success or failure of health information systems depends upon a match between the system demands and the end user’s level of electronic health literacy [59]. At the same time, it’s necessary to take into account that scales used in most studies are not objective measures. Sometimes self-evaluation could skew the findings as self-evaluated digital competencies do not translate into the efficacy of computer use [56]. As the relationship between self- perception and actual behavior is often weak, it would be important to ask not only for self-report about a skill but also skill demonstration as well [61]. At the same time, it’s necessary to consider that in some studies [20] the scales used were not validated and that none of the instruments had a specific cut-off to define poor digital literacy.

The complexity of the interaction between digital literacy, health literacy and health outcomes as well as the design of the studies reviewed, mostly voluntary surveys or interviews, did not allow for a strong identification of the effects of digital literacy on the outcomes for specific pathologies. Guo et al [51] aimed to demonstrate the relationship between eHealth literacy (eHL), mobile health literacy (mHL), and health outcomes, particularly HbA1c, in a sample of Taiwanese patients with type 2 diabetes. The study found that mobile eHL had a direct effect on self-care behavior as well as knowledge and skills of computers, the internet, and mobile technology, and had an indirect effect on health outcomes (glycemic control and self-rated health status). In statistical terms, higher mobile eHL cannot be assumed to reduce HbA1c in this study.

Instead, most general consequences could be identified according to the patient´s perspectives like decreased access to health care portals, increased wait time for medical appointments, inappropriate use of emergency services, and preventative care coordination. In this sense, poor digital literacy has the potential to worsen most healthcare outcomes.

At the same time, several authors propose that higher digital literacy could be correlated with better quality of life, health promotion and mental health [2,55,63,64,66,69]. G Kim et al [66] found that eHealth literacy was the strongest predictor of health behaviors after adjusting for sociodemographic and health-related characteristics. These findings indicate that eHealth literacy can be an important factor in promoting individual health behaviors.

Although most of the analyzed publications propose different strategies to improve digital literacy, we could find only 7 studies that reported interventions addressing the topic. These interventions can be divided into 2 categories: provision of education and training, and social support. Under education and training, massive open online courses, university training in e-health through tutoring, and online video-based portal training were reported to improve digital health literacy of both children and adults. On the other hand, social support from technology-savvy family members, professionals, and peers were shown to improve digital health literacy of adults and older adults.

According to the patient´s perspectives, instruction needs to be simple particularly for older adults or individuals with cognitive impairments such as memory loss. Patients recommended educational institutions such as universities and health care organizations as good venues for hosting workshops, ongoing classes, and even a dedicated technology help desk in clinics where patients and family members could learn how to navigate their online patient portals in-person, conduct a video visit, and use technology in general. Additionally, synchronous (eg, a phone line) and asynchronous (eg, preparatory instructional videos and written information) remote learning resources can help patients overcome technology issues related to video visits or the use of patient portals. Several patients also envisioned having technology champions and coaches directly in their community. In-person training was preferred because it was considered more relatable and easier to understand. Surprisingly, scarce literature is available that evaluates interventions based on the patient´s perspectives.

In order to address the impact of digital divide on overall health outcomes, we need to gain greater knowledge about patients´digital health literacy and design strategies based on their perceived needs. Ensuring technology access is only 1 facet of the divide, improved technology design and training are critical for improving patients’ digital health literacy. Future work should be focused on better quality studies that could assess digital literacy based on objective measures of skills and not only on the patient’s self rated measures.

Strategies to improve digital literacy should be designed taking into account patients’ perspectives and their effect needs to be evaluated in high quality research that could measure not only the initial improvement but also its sustainability over time.

## Conclusions

Digital health information resources and digital interaction with providers have great advantages with the potential to improve the efficiency, quality and reach of healthcare systems while empowering the patient. However, it is very important that commitment to this strategy leaves no one behind. Ethnic minorities, the elderly and patients of low socioeconomic status are at risk of having low digital literacy and, therefore, of having increasing difficulty accessing healthcare as the wave of digital health unfolds.

Increased interest in this issue in the form of publications is a good starting point but there is a need to improve measurement tools, broaden the geographic diversity of studies, as well as insist on the creation, deployment and validation of interventions aimed at reducing poor digital health literacy.

## Supporting information

S1 PRISMA Checklist PRISMA Checklist. (XLSX)

S1 Text Search Strategy: Master Medline (Ovid) strategy. (DOCX)

S1 Template Template of the standardized data collection form. (XLSX)

S1 Data Data used to construct Figs 1 and 2. (XLSX)

S1 Table Summary of included studies. (DOCX)

S2 Table Concepts related to digital health literacy and corresponding theoretical framework in the included studies. (DOCX)

S3 Table Assessment tools for digital health literacy. (DOCX)
