# Supplementary material for: Digital literacy as a new determinant of health: A scoping review
Source: PLOS Digit Health. 2023 Oct 12;2(10):e0000279. doi: 10.1371/journal.pdig.0000279 (PMC10569540; doi:10.1371/journal.pdig.0000279)
Supplement: S1 Text — (DOCX) [file pdig.0000279.s002.docx]

**Digital determinants of health**

**Digital literacy as a new determinant of health: a scoping review**

**S1 Text:** **Master Medline (Ovid) strategy**
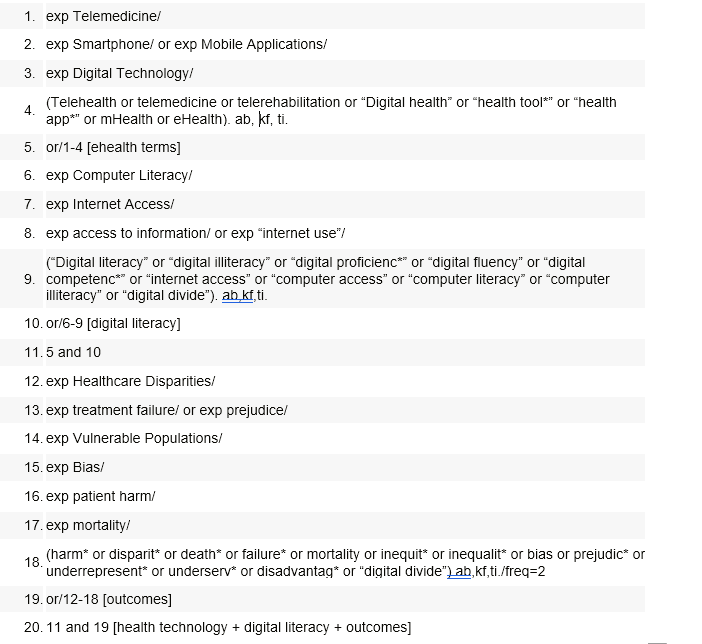


Terms denoted with a “/” are controlled vocabulary terms (from term thesauruses such as MeSH or Emtree) that are assigned to articles when they are indexed into the literature databases.  Search lines with the tags “ab, kf, ti” are natural language searches within the article abstract, keywords, or title.  By combining controlled vocabulary and natural language terms, we are able to cast a wider net than if we used one or the other only.  Terms in brackets are not commands, but rather search line labels to aid in strategy interpretation. The master Medline (Ovid) strategy was also translated and run in Embase (Ovid), Scopus, and Google Scholar
