# Supplementary material for: Digital literacy as a new determinant of health: A scoping review
Source: PLOS Digit Health. 2023 Oct 12;2(10):e0000279. doi: 10.1371/journal.pdig.0000279 (PMC10569540; doi:10.1371/journal.pdig.0000279)
Supplement: S1 Table — (DOCX) [file pdig.0000279.s005.docx]

**Digital determinants of health**

**Digital literacy as a new determinant of health: a scoping review**

## **S1 Table. Summary of included studies**

| **Author, Year** | **Journal** | **Article type** | **Objective** | **Setting (Country)** | **Study population (sample size)** | **Tackled concept related to digital health literacy (Author of conceptual framework)** | **Assessment Tool** | **Health outcomes affected by digital health literacy** | **Interventions targeted to reduce digital health literacy gap** |
| --- | --- | --- | --- | --- | --- | --- | --- | --- | --- |
| Abdulai et al  2021 ^[57]^ | International Journal of Medical Informatics | Cross-sectional study | To examine the digital literacy of lay consumers of online COVID-19-related information in Ghana. | Ghana | online health consumers (n=325) | eHealth literacy ^[23]^ | eHEALS |  | Intergenerational help from technology-savvy family members such as children and grandchildren |
| Adil et al,  2021 ^[28]^ | BMC Public Health | Cross-sectional study | To probe the relationship of education and Institution [Independent Variables] with the usage and expertise in eHealth literacy [Dependent Variables] among university students. | Pakistan | students in 16 universities (n=1,513) | Digital health literacy ^[23]^ | eHEALS |  |  |
| Alkureishial.,  2021 ^[2]^ | JMIR human factors | Cross-sectional study | To understand patient´s perspectives on [1] the definition, causes, and impact of the digital divide; [2] whose responsibility it is to address this divide, and [3] potential solutions to mitigate the digital divide. | USA | adult patients and parents of pediatric patients who had virtual visits at the University of Chicago Medical Center primary care clinics (n=54 interviews) | Digital literacy ^[23]^ |  | Health promotion, Quality of life |  |
| Alvarez-Perez et al., 2021 ^[20]^ | JMIR Diabetes | Mix methods study | To develop MOOCs designed to improve the digital health literacy level of people with type 1 diabetes [T1D] and type 2 diabetes [T2D] in 5 European countries [Spain, Belgium, Denmark, Italy, and Sweden] under the framework of the IC-Health project | Spain, Belgium, Denmark, Italy, and Sweden | adults and adolescents with diabetes (n=28) | Digital health literacy ^[23]^ |  |  | Massive open online courses |
| Aponte and Nokes, 2017 ^[62]^ | Journal of clinical nursing | Cross-sectional study | To examine the validity of the Spanish version of an instrument used to measure electronic health literacy [eHEALS] with an older Hispanic population from a number of Spanish - language countries living in New York City in the United States. | USA | senior organization (n=100) | Electronic health literacy ^[23]^ | eHEALS |  |  |
| Arcury et al., 2020 ^[58]^ | Journal of Applied Gerontology | Cross-sectional study | [1] To determine Internet use, and to identify computer characteristics, social support, general health knowledge and attitudes, health status, and personal characteristics associated with Internet use; [2] To identify the levels of eHealth literacy using the eHEALS scale, and to identify computer and Internet characteristics, social support, general health knowledge and attitudes, health status, and personal characteristics associated with eHealth literacy level. | USA | minority [African American, Latino, American Indian, Asian] and White older adult patients (n=200) | eHealth literacy ^[23]^ | eHEALS |  |  |
| Berkowsky, 2020 ^[71]^ | Gerontology and Geriatric Medicine | Cross-sectional study | To identify disparities in eHealth literacy among older adults aged 65+ residing in California, USA | USA | Older adults aged 65+ (n=237) | eHealth literacy ^[23]^ | eHEALS |  |  |
| Chen, Elsworth, and Osborne  2020 ^[21]^ | Frontiers in public health | Mix methods study | To apply the Ophelia [Optimizing Health Literacy and Access] process, a widely used systematic approach to whole of community co-design, to the digital context to generate solutions to improve health and equity outcomes. | Australia | Adult patients in 3 health sites in Australia (n=530) | eHealth literacy ^[23]^ | eHLQ |  |  |
| Cherid et al., 2020 ^[80]^ | Osteoporos Int | Cross-sectional study | To identify the current level of technology adoption, health, and eHealth literacy among older adults with a recent fracture, to determine if the use of electronic interventions would be feasible and acceptable in this population. | Canada | Adults 50 years and older who recently suffered a fracture (n=401) | eHealth literacy ^[23]^ | eHEALS |  |  |
| Chun et al., 2022 ^[47]^ | International Journal of Environmental Research and Public Health | Cross-sectional study | To adapt Digital Health Literacy Instrument into Korean and to assess the psychometric properties, during the COVID-19 pandemic period. | South Korea | Korean undergraduates (n=604) | Digital health literacy | COVID-DHL-K |  |  |
| Chung et al., 2018 ^[66]^ | Journal of Medical Internet Research | Cross-sectional study | To develop a Korean version of eHEALS and evaluate its reliability and validity employing healthy young adults in Korea. | South Korea | Registrants of a Korean internet survey panel service agency (n=500) | eHealth literacy ^[23]^ | eHEALS |  |  |
| Hoz et al., 2021 ^[17]^ | International Journal of Environmental Research and Public Health | Quasi-experimental longitudinal study | To determine the level of eHealth literacy, scientific knowledge in health and confidence on the part of students, analyzing the degree of improvement following a teaching intervention with students of the Bachelor’s Degree of Education of the University of Extremadura. | Spain | University students (n=42) | eHealth literacy ^[23]^ | eHEALS |  | University training in eHealth through interventions based on a cooperative active methodology [tutoring] |
| Frings et al., 2022 ^[13]^ | BMC Public Health | Cross-sectional study | To investigate university students’ digital health literacy and web-based information-seeking behaviours during the early stages of the COVID-19 pandemic in England. | United Kingdom | University students (n=691) | Digital health literacy ^[23]^ | DHLI |  |  |
| Fuzhi W et al., 2019 ^[50]^ | SAGE Open | Cross-sectional survey | To evaluate the health information literacy [HIL] level and influencing factors among digital immigrants in rural China and to investigate their obstacles of online health information seeking. | China | Adult residents (n=1,132) | Health information literacy | Modified EHIL-10 |  |  |
| Gillie et al., 2022 ^[27]^ | Journal of Alzheimer's Disease Reports | Cross-sectional study | To propose the Telehealth Literacy Screening Tool [TLST] for use in older adults and support the future inclusion of telehealth literacy as an important social determinant of health [SDOH] | USA | Older adults and low-health literate patients at a clinic (n=90) | Telehealth literacy ^[27]^ | TLST |  |  |
| Guo SMH et al., 2021 ^[51]^ | JMIR mHealth and uHealth | Cross-sectional study | To present data about online information-seeking behavior and mobile health [mHealth] app usage, investigate the factors related to mobile eHL in Taiwanese patients with type 2 diabetes, and flesh out the relationship between eHealth literacy [eHL], mobile health literacy [mHL], and health outcomes. | Taiwan | Taiwanese patients with type 2 diabetes (n=249) | eHealth literacy ^[23]^  mHealth literacy ^[25]^ | Mobile eHealth Literacy Questionnaire | Disease prevalence |  |
| Guo Z et al., 2021 ^[63]^ | Journal of Medical Internet Research | Cross-sectional study | To evaluate socioeconomic disparities in eHealth literacy and seeking of web-based information on COVID-19, and their associations with COVID-19 preventive behaviors. | Hong Kong | Adults (n=1,501) | eHealth literacy ^[23]^ | eHEALS | Health status |  |
| Hannemann et al., 2021 ^[73]^ | BMC Medical Informatics and Decision Making | Cross-sectional study | To explores how the use of digital health technologies, which connect patients with health care providers and health insurers has changed during the COVID-19 pandemic, | Germany | Adults (n=1,570) | Digital literacy | Modified eHEALS |  |  |
| Hoogland et al., 2020 ^[59]^ | Journal of Geriatric Oncology | Cross-sectional study | To examine age differences in eHealth literacy and use of technology devices/HIT in patients with cancer, and characterize receptivity towards using home-based HIT to communicate with the oncology care team | USA | Patients in a radiation oncology clinic (n=198) | eHealth literacy ^[23]^ | eHEALS |  |  |
| Hussein et al., 2021 ^[26]^ | Clinical Journal of the American Society of Nephrology | Cross-sectional study | To assess the availability of devices and the internet, proficiency, and interest in using mobile health | USA | Hemodialysis and home dialysis patients (n=949) | Mobile health proficiency | - |  |  |
| Hyman et al., 2020 ^[18]^ | Preventive Medicine Reports | Pre-post test study | To test the effectiveness of a novel intervention to increase students’ digital health literacy and health knowledge | Canada | Intermediate elementary students (n=126 at pre-intervention, n=119 at post-intervention, n=104 at 2-month follow-up) | Digital health literacy ^[23]^ | eHEALS |  | Learning for Life [L4L] school-based intervention |
| Ismond et al., 2021 ^[42]^ | Journal of medical systems | Cross-sectional study | To characterize the readiness of patients with cirrhosis for e-health by: [1] assessing their Internet access frequency and digital technology ownership; [2] determining their digital literacy proficiency and identifying relevant predictors; and, [3] ascertaining their general attitudes and receptiveness to video conferencing and online health management programs by age group | Canada | Patients with cirrhosis (n=117) | Digital literacy | Computer Proficiency Questionnaire, and the Mobile Device Proficiency Questionnaire |  |  |
| Jang et al., 2022 ^[70]^ | International Journal of Advanced and Applied Sciences | Cross-sectional study | To identify the correlation between digital literacy, loneliness, quality of life, and health promotion behaviors among the elderly aged between 65 and over and under 75 years | South Korea | Elderly [65-75 y/o] (n=159) | Digital literacy | Digital literacy tool ^[40]^ | Quality of life |  |
| Jun et al.  2020 ^[30]^ | International Journal of Environmental Research and Public Health | Cross-sectional study | To identify [1] that the digital divide of the older people group is more serious than that of the other information-weak groups [disabled people, farmers/ fishermen, and low-income and [2] the priorities that affect the digital divide among the three elements of the digital divide [access, capacity, and utilization]. | South Korea | 4 subgroups: [1] Disabled people, [2] low-income people, [3] farmers and fisherman, [4] older people [n unspecified] | Digital capability level | PC usability and mobile device usability |  |  |
| Kayser et al., 2019 ^[33]^ | Journal of Medical Internet Research | Cross-sectional study | To evaluate how the eHealth Literacy Questionnaire [eHLQ] combined with selected dimensions from the Health Education Impact Questionnaire [heiQ] and the Health Literacy Questionnaire [HLQ] can be used together as an instrument to characterize an individual’s level of health technology readiness and explore how the generated data can be used to create health technology readiness profiles of potential users of health technologies and digital health services | Denmark | Patients with a recent cancer diagnosis referred to rehabilitation (n=305) | eHealth literacy ^[24]^ | READHY tool |  |  |
| Kemp et al., 2021 ^[34]^ | Health Promotion Journal of Australia | Cross-sectional study | To examine consumer and other stake-holder views on implementation of digital health approaches in can-cer care in Australia | Australia | Consumers, health care professionals, researchers, developers, nongov-ernment and government/policy stakeholders (n=51) | eHealth literacy ^[24]^ |  | Disease prevalence, Health status |  |
| Kim H et al., 2021 ^[52]^ | International Journal of Older People Nursing | Cross-sectional study | To compare the psychometric properties of two measures of eHealth literacy, namely, the Korean versions of the Digital Health Literacy Instrument [K-DHLI] and eHealth Literacy Scale [K-eHEALS], among older adults in South Korea | South Korea | Older adults [65 y/o and above] (n=180) | eHealth literacy ^[23]^ | DHLI, eHEALS |  |  |
| Kim SH et al., 2017 ^[67]^ | Computers, informatics, nursing | Cross-sectional study | To examine the relationship between eHealth literacy and self-reported health behaviors in Korean adults | South Korea | Young Korean Internet users [18-39 y/o] (n=230) | eHealth literacy ^[23]^ | eHEALS | Health promotion |  |
| Leung et al., 2022 ^[53]^ | Aging and Mental Health | Cross-sectional study | To analyze the relationships of sense of coherence with anxiety, digital health literacy [DHL], information, and financial satisfaction among older adults during the outbreak. | China, Philippines, Singapore | Older adults [at least 60 y/o] (n=266) | Digital health literacy ^[23]^ | DHLI | Mental and psychological states |  |
| Li SJ et al., 2020 ^[64]^ | International Journal of Environmental Research and Public Health | Cross-sectional study | To examine the associations among health-promoting lifestyles, eHealth literacy, and cognitive health in older adults | China | Chinese older adults [at least 60 y/o] (n=1,201) | eHealth literacy ^[23]^ | eHEALS | Health promotion |  |
| Li X et al., 2020 ^[65]^ | Journal of Medical Internet Research | Cross-sectional study | To explore the predictive role of social media use on public preventive behaviors in China during the COVID-19 pandemic and how disease knowledge and eHealth literacy moderated the relationship between social media use and preventive behaviors. | China | Chinese internet users (n=802) | eHealth literacy ^[23]^ | eHEALS | Health promotion |  |
| Liu et al.  2020 ^[46]^ | Journal of Medical Internet Research | Cross-sectional study | To create an instrument for digital health literacy assessment [DHLA] based on the eHealth Literacy Scale [eHEALS] to categorize participants by level of risk of misinterpreting health information into high-, medium-, and low-risk groups. | Taiwan | Taiwanese residents 20 y/o and above (n=350) | eHealth literacy ^[23]^ | DHLA |  |  |
| Lyles et al., 2019 ^[15]^ | Journal of the American Board of Family Medicine | Randomized controlled trial | To test the effectiveness of delivering online, video-based portal training to patients in a safety net setting | USA | English-speaking adults diagnosed with chronic condition (n=93) | eHealth literacy ^[23]^ | eHEALS |  | Online video-based portal training |
| Magsamen-Conrad et al., 2019 ^[32]^ | Health communication | Cross-sectional study | To identify: [1] how do middle-aged and older adults use technology to seek health information?, [2] do middle-aged and older adults communicate with others about health and technology? [3] What role does literacy play in the process of using technology to seek health information? | USA | Middle-aged and older adults (n=55) | Technological literacy New media literacy, eHealth literacy ^[81,22]^ | - |  |  |
| Martínez-Alcalá et al., 2021 ^[19]^ | Frontiers in Education | Pre-post test study | To analyze the level of Digital Literacy with the Digital Literacy Evaluation [DILE] of two groups of elderly adults with different levels of literacy. | Mexico | Older adults aged around 60, and with a range of 12–14 years of education (n=176) | Digital literacy | DILE |  | Blended digital literacy workshop |
| Moon et al., 2022 ^[60]^ | Supportive Care in Cancer | Cross-sectional study | To explore e-health literacy rates and access to smartphones and tablets in a large sample of breast cancer survivors. | UK | Breast cancer survivors (n=2,009) | eHealth literacy ^[23]^ | eHEALS |  |  |
| Nguyen et al., 2021 ^[55]^ | International journal of environmental research and public health | Cross-sectional study | To examine the association between the fear of COVID-19 scale and the health related quality of life, and to examine the effect modification by HL, eHEAL, and DDL on this association. | Vietnam | Adults (n=4,348) | eHealth literacy ^[23]^  digital healthy diet literacy ^55]^ | eHEALS, DDL-4 questionnaire | Quality of life |  |
| Nouri et al., 2019, ^[31]^ | JMIR mHealth and uHealth | Cross-sectional study | To describe variations in patients' engagement in the app design process, focusing on limited health literacy [LHL], limited English proficiency [LEP], and limited digital literacy [LDL] | USA | Patients from primary care clinics (n=20) | Digital literacy ^[82,23]^  Mobile phone digital literacy | - |  |  |
| Papp-Zipernovszky et al., 2021 ^[35]^ | Frontiers in Public Health | Cross-sectional study | To explore generational differences in IHISB and digital HL first in Hungary, as well as a self-rated and a more objective application of these skills [perceived empowerment and health care utilization]. | Hungary | Four generations in Hungary (n=522) | eHealth literacy or digital health literacy ^[38, 23, 24]^ | eHEALS | Quality of life |  |
| Patil et al.  2021 ^[54]^ | International Journal of Environmental Research and Public Health | Cross-sectional study | To explore the associations of health information access and sources, as well as COVID-19-related attitudes and behaviors, with HL and DHL in U.S. college students. | USA | College students (n=256) | Digital health literacy ^[23]^ | DHLI |  |  |
| Schrauben et al., 2021 ^[61]^ | American Journal of Kidney Diseases | Cross-sectional study | To describe the use of mHealth technologies, as well as willingness to use mHealth technologies and the level of eHealth literacy among individuals with mild-to-moderate CKD in the Chronic Renal Insufficiency Cohort [CRIC] Study. | USA | Patients with chronic renal insufficiency (n=932) | eHealth literacy ^[23]^ | eHEALS |  |  |
| Shiferaw et al., 2020 ^[29]^ | BMC health services research | Cross-sectional study | To assess digital competency of healthcare providers among seven public health centers in North-West Ethiopia. | Ethiopia | healthcare providers working in seven public health centers [n=193] | Digital competency ^[83]^ | Digital competency questionnaire |  |  |
| Shiferaw et al., 2020 ^[74]^ | BMC medical informatics and decision making | Cross-sectional study | To assess eHealth literacy level and associated factors among internet user chronic patients in North-west Ethiopia. | Ethiopia | internet user chronic patients (n=423) | eHealth literacy ^[23]^ | eHEALS |  |  |
| Tsai et al.,  2017 ^[16]^ | Journal of applied gerontology : the official journal of the Southern Gerontological Society | Randomized controlled trial | To understand how older adults learn to use a specific technology, tablet computers, and the role that social support plays in this process. | USA | older adults aged 69 to 91 years old (n=309) | Digital literacy | Semi-structured and in-depth interviews |  | Support from three areas: family, professionals, and peers. |
| van der Vaart et al., 2019 ^[22]^ | JMIR Formative Research | Mixed methods study | To assess the usability of an iCBT for chronic pain, Master Your Pain, and the relationship between its usability outcomes and the factors age, educational level, and digital health literacy skills. | Netherlands | Patients from mental health care institutions (n=32) | eHealth literacy or digital health literacy ^[23]^ | DHLI |  |  |
| Yang et al., 2021 ^[69]^ | Journal of Nursing Management | Cross-sectional study | To investigate the eHealth literacy and the psychological status of Chinese residents during the COVID-19 pandemic and explore their interrelationship. | China | Chinese residents (n=15,000) | eHealth literacy ^[23]^ | eHLQ | Mental and psychological states |  |
| Yun et al., 2022 ^[14]^ | European Journal of Internal Medicine | Randomized controlled trial secondary analysis | To determine the effectiveness of a telemedicine [TM]-based managed care solution across literacy levels and information and communications technology [ICT] skills. | Spain | Patients with heart failure (n=178) | eHealth literacy ^[23]^ | eHEALS [computer literacy and traditional literacy domains only] |  |  |
| Perestelo-Perez et al., 2020 ^[75]^ | Sustainability | Cross-sectional study | To present the objectives, activities and results of the IC-Health project whose objective was to develop a series of massive open online courses [MOOCs] to improve the DHL skills of European citizens. | Spain, Italy, Belgium, the United Kingdom, Sweden, Denmark and Estonia | Adults (n=862), children (n=384), adolescents (n=396) | Digital health literacy ^[23]^ | eHEALS | Health promotion, Disease prevalence | Series of massive open online courses |
| Duong et al., 2020 ^[56]^ | International Journal of Environmental Research and Public Health | Cross-sectional study | To examine the psychometric properties of digital healthy diet literacy [DDL] and its association with eating behavior changes during the COVID-19 pandemic among nursing and medical students. | Vietnam | Undergraduate students (n=7,616) | Digital healthy diet literacy | DDL |  |  |
| Paige et al., 2020  ^[37]^ | Journal of Health Communication | Cross-sectional study | To describe the psychometric testing of a multi-dimensional instrument to measure functional, communicative, critical, and translational eHealth literacies, as informed by the Transactional Model of eHealth Literacy [TMeHL]. | USA | Phase 1: end-users and experts (n=30); Phase 2: end-users (n=10); Phase 3: patients from university-based registry (n=283) | Transactional eHealth literacy ^[38]^ | TeHLI |  |  |
| Jean et al., 2017 ^[48]^ | Journal of Consumer Health on the Internet | Cross-sectional study | To design and implement an after-school program [HackHealth] that aims to increase tweens’ [i.e., young people approaching or at the beginning of their teen years] interest in science and health, improve their digital health literacy skills, and increase their health-related self-efficacy. | USA | Middle school students (n=33) | Digital health literacy | DHLAT |  | After-school program [HackHealth] |
| Seckin et al., 2016 ^[12]^ | Journal of Medical Internet Research | Cross-sectional study | To examine psychometric properties of a new electronic health literacy [ehealth literacy] measure in a national sample of Internet users with specific attention to older users. | USA | Respondents who used the Internet for health information (n=710) | Digital health literacy | e-HLS |  |  |
| Karnoe et al., 2018 ^[36]^ | Journal of Medical Internet Research | Cross-sectional study | To develop and validate an eHealth literacy assessment toolkit [eHLA] that assesses individuals’ health literacy and digital literacy using a mix of existing and newly developed scales. | Denmark | Adults from outpatient and community (n=475) | eHealth Literacy ^[23, 24]^ | e-HLA |  |  |

COVID-DHL-K: Digital Health Literacy Instrument in Relation to COVID-19 Information; DDL: Digital Healthy Diet Literacy; DHLA: Digital Health Literacy Assessment

DHLAT: Digital Health Literacy Assessment Tool; DHLI: Digital Health Literacy Instrument; DILE: Digital Literacy Evaluation

eHEALS: eHealth Literacy Scale ; EHIL-10: Everyday Health Information Literacy–10

eHLA: eHealth Literacy Assessment ; EHLQ: eHealth Literacy Questionnaire; e-HLS: electronic Health Literacy Scale

READHY: Readiness and Enablement Index for Health Technology Tool ; TLST: Telehealth Literacy Screening Tool; TeHLI: transactional eHealth literacy instrument

y/o: years old

**References**

All the references are in the main text of the manuscript except the following

80. Cherid C, Baghdadli A, Wall M, Mayo N, Berry G, Harvey E et al. Current Level of Technology Use, Health and Ehealth Literacy in Older Canadians with a Recent Fracture—a Survey in Orthopedic Clinics. Osteoporosis International*.* 2020; 31[7]: 1333-1340; doi:10.1007/s00198-020-05359-3

81. Berkman N, Davis T, McCormack L. Health Literacy: What Is It? Journal of Health Communication. 2010;15[sup2]:9-19.

82. Mamedova S, Pawlowski E. A Description of U.S. Adults Who Are Not Digitally Literate. U.S. Department of Education; 2018 p. 2,19

83. Joint Research Centre, Institute for Prospective Technological Studies, Punie Y, Ferrari A, Brečko B. DIGCOMP – A framework for developing and understanding digital competence in Europe. Publications Office; 2013. Available from: doi/10.2788/52966
