# Supplementary material for: Digital literacy as a new determinant of health: A scoping review
Source: PLOS Digit Health. 2023 Oct 12;2(10):e0000279. doi: 10.1371/journal.pdig.0000279 (PMC10569540; doi:10.1371/journal.pdig.0000279)
Supplement: S2 Table — (DOCX) [file pdig.0000279.s006.docx]

**Digital determinants of health**

**Digital literacy as a new determinant of health: a scoping review**

## **S2 Table. Concepts related to digital health literacy and corresponding theoretical framework in the included studies**

| **Terms related to digital health literacy** | **Definition** | **Theoretical framework(s) or model(s)** | **Reference** |
| --- | --- | --- | --- |
| Digital literacy | Adults were defined as “not digitally literate” using the following requirements for basic computer competence: [1] prior computer use, [2] willingness to take the assessment on the computer, and [3] passing a basic computer test [by successfully completing four of six simple tasks, such as using a mouse and highlighting text on the screen]. Adults who did not meet any one of these requirements or did not take the digital problem-solving assessment are classified as not digitally literate in this Brief . | “...to operate effectively in today’s digital environment, one needs to master foundational computer skills, including [a] skills associated with manipulating input and output devices [e.g., the mouse, the keyboard, and digital displays], [b] awareness of concepts and knowledge of how the digital environment is structured [e.g., files, folders, scrollbars, hyperlinks, and different types of menus and buttons], and [c] the ability to interact effectively with digital information [e.g., how to use commands such as Save, Delete, Open, Close, Move, Highlight, Submit, and Send]” | US Department of Education ^[82]^ |
| Digital competency or digital information literacy | “the set of knowledge, skills, attitudes, abilities, strategies, and awareness that are required when using ICT and digital media to perform tasks; solve problems; communicate; manage information; collaborate; create and share content; and build knowledge effectively, efficiently, appropriately, critically, creatively, autonomously, flexibly, ethically, reflectively for work, leisure, participation, learning, and socializing” | Digital competency framework has five core digital literacy components: [1] Information/data literacy, [2] content creation, [3] communication, [4] problem solving and [5] safety | European Union Commission ^[83,84]^ |
| Health literacy | “the degree to which individuals can obtain, process, understand, and communicate about health-related information needed to make informed health decisions” ^[14]^ |  | Berkman et al. ^[81]^ |
| Digital health literacy [DHL] | “...requires a unique skill set, including the ability to find, evaluate, appraise, integrate, and apply health information from online environments” | [1] operational skills; [2] navigation skills; [3] information searching; [4] evaluating reliability; [5] determining relevance; [6] adding content; [7] protecting the privacy ^[30]^ | Patil et al. ^[54]^ |
| eHealth literacy | “...the ability to seek, find, understand, and appraise health information from electronic sources and apply the knowledge gained to addressing or solving a health problem” | **Lily model**. At the heart of eHealth literacy are six core skills [or literacies]: traditional literacy, health literacy, information literacy, scientific literacy, media literacy, and computer literacy. Likened to a lily flower, the petals [literacies] feed the pistil [eHealth literacy], and yet the pistil overlaps the petals, tying them together. | Norman and Skinner ^[23]^ |
|  |  | **e-health literacy framework**. eHealth literacy has seven domains: [1] Ability to process information, [2] Engagement in own health, [3] Ability to engage actively with digital services, [4] Feeling safe and in control, [5] Motivation to engage with digital services, [6] Having access to systems that work, and [7] Digital services that suit individual needs | Norgaard et al. ^[24]^ |
| mHealth literacy | “...the ability to use mobile devices to search, find, understand, appraise, and apply health information to address or  solve a health problem” | **mHealth literacy** has two dimensions based on the required skills of using mobile devices: [1] health-related information seeking or the capability to search and find health information using mobile devices and [2] health information appraisal or the ability to understand, appraise, and apply health information in mobile devices. | Lin and Bautista ^[25]^ |
| mobile health proficiency | Various elements of internet or device users were converted to three levels of proficiency: basic use [phone calls and text messages only], intermediate use [video calls, email, photos, videos, online media, social media, internet articles/news, alarms, reminders, maps, directions, or calendar], and advanced use [internet shopping, personal health information, planning and tracking, booking travel or entertainment, banking, or online payments]. The nonproficient group comprised basic users and nonusers, and the proficient group comprised intermediate and advanced users. |  | Hussein et al. ^[26]^ |
| telehealth literacy | “...a combination of elements of technologic and health literacy that allows for a patient to access, enable, and navigate their telehealth platform” |  | Gillie et al. ^[27]^ |
| mobile phone digital literacy | “...participants with very limited use or engagement and/or self-reported difficulty in using their mobile phones as having LDL [limited digital literacy]” |  | Nouri et al. ^[31]^ |
| digital capacity | “...digital capacity level consists of two elements, that is, PC usability and mobile device usability, respectively. PC usability is the ability to indicate whether basic activities are possible using a PC. On the other hand, mobile device usability means the ability to indicate whether basic activities are possible using mobile devices, including smartphones.” |  | Jun ^[30]^ |
| technology and technological literacy | “the ability to responsibly use appropriate technology to communicate, solve problems, and access, manage, integrate, evaluate, and create information to improve learning in all subject areas and to acquire lifelong knowledge and skills in the 21st century” |  | State Educational Technology Directors Association ^[85]^ |
| new media literacy | “the ability to critically utilize technology and new media to accomplish a goal” |  | Magsamen-Conrad ^[32]^ |
| digital healthy diet literacy [DDL] | “...the ability to access, understand, judge, and apply digital healthy-diet-related information to improve healthy eating behavior and health outcomes” | Health literacy conceptual framework was expanded to assess the information processing ability to [1] find reliable and accurate healthy diet information on the internet, [2] understand healthy diet information and dietary guidelines on the internet, [3] judge whether healthy diet information on the internet is applied for individuals, and [4] apply healthy diet information from the internet into individuals’ daily lives to eat healthily. | Duong et al. ^[49]^ |

## References:

All references are in the main text of the manuscript except the following

81. Berkman N, Davis T, McCormack L. Health Literacy: What Is It? Journal of Health Communication. 2010;15[sup2]:9-19.

82. Mamedova S, Pawlowski E. A Description of U.S. Adults Who Are Not Digitally Literate. U.S. Department of Education; 2018 p. 2,19

83. Joint Research Centre, Institute for Prospective Technological Studies, Punie Y, Ferrari A, Brečko B. DIGCOMP – A framework for developing and understanding digital competence in Europe. Publications Office; 2013. Available from: doi/10.2788/52966

84. Ferrari A. Digital Competence in Practice: An Analysis of Frameworks [Internet]. Luxembourg: Publications Office of the European Union; 2012 p. 3-4. Available from: <https://ifap.ru/library/book522.pdf>

85. State Educational Technology Directors Association. 2007 Technology Literacy Assessment and Educational Technology Standards Report. 2007.
