# Supplementary material for: Digital literacy as a new determinant of health: A scoping review
Source: PLOS Digit Health. 2023 Oct 12;2(10):e0000279. doi: 10.1371/journal.pdig.0000279 (PMC10569540; doi:10.1371/journal.pdig.0000279)
Supplement: S3 Table — (DOCX) [file pdig.0000279.s007.docx]

**Digital determinants of health**

**Digital literacy as a new determinant of health: a scoping review**

## **S3 Table. Assessment tools for digital health literacy**

| **Assessment Tool** | **Author** | **Elements** | **Aim / Intended use** | **Mode** | **Scoring** | **Language** | **Reliability, Cronbach α** | **Included studies that used the measurement tool** |
| --- | --- | --- | --- | --- | --- | --- | --- | --- |
| Digital Healthy Diet Literacy [DHDL] | Duong et al. ^[49]^ | ability to [1] find reliable and accurate healthy diet information on the internet, [2] understand healthy diet information and dietary guidelines on the internet, [3]] judge whether healthy diet information on the internet is applied for individuals, and [4]] apply healthy diet information from the internet into individuals’ daily lives to eat healthily | “...for the quick assessment of students’ ability to access, understand, appraise, and apply healthy diet information found on the internet” | self-rated | 4-point Likert scale | Vietnamese | nursing and medical students, Vietnam: 0.86 ^[49]]^ | ^[55]^ |
| Digital Health Literacy Assessment [DHLA] | Liu et al. ^[46]^ | [1]] self-assessment of digital health literacy; [2]] how convincing people found internet health information from different sources; [3] trust in health information from folklore and customs | “...to categorize participants by level of risk of misinterpreting health information into high-, medium-, and low-risk groups” | self-rated | 10-item, 5-point Likert scale | Chinese | Taiwanese residents aged 20 years and older: 0.87 ^[46]]^ | ^[46]^ |
| Digital Health Literacy Assessment Tool [DHLAT] | Jean et al. ^[48]^ | [1] functional health literacy; [2] health literacy self-assessment; [3] familiarity with health and health care; [4] knowledge of health and disease; [5] technology familiarity; [6] technology confidence; [7] incentives for engaging with technology | “...to assess participants’ pre-existing digital health literacy skills and to identify specific skills on which they might benefit from further instruction” | story-based, self-administered questionnaire | qualitative | English | - | ^[48]^ |
| Digital Health Literacy Instrument [DHLI] | van der Vaart and Drossaert ^[45]^ | [1] operational skills; [2] navigation skills; [3] information searching; [4] evaluating reliability; [5] determining relevance; [6] adding content; [7] protecting the privacy | “To measure the complete spectrum of Health 1.0* and Health 2.0** skills, including actual competencies…” | self-  rated and performance-based | self-rated: 21-item, 4-point Likert scale  performance-based: 7-item | Danish | adult general Dutch population, Netherlands: 0.87 for self-report, 0.47 for performance-based items, ^[30]^ | ^[22]^ |
|  |  |  |  |  |  | English | university students, England: 0.86 ^[13]^  undergraduate and postgraduate non-healthcare and healthcare students, England: 0.51 ^[13]^ | ^[13]^ |
|  |  |  |  |  |  | Korean | Older adults, South Korea: 0.77 ^[52]^ | ^[52]^ |
|  |  |  |  |  |  | Chinese and English | Older adults, China, Philippines, Singapore: 0.91 | ^[53]^ |
| Digital Health Literacy Instrument in Relation to COVID-19 Information [COVID-DHL-K] | Chun et al. ^[47]^ | [1] Searching the web for information on coronavirus;  [2] Adding self-generated content on coronavirus;  [3] Evaluating the reliability of coronavirus-related information;  [4] Determining personal relevance of coronavirus-related information;  [5] Protecting privacy on the Internet | “...valuable in the development of related policy aiming to increase digital health literacy and compliance with the policies meant to control COVID-19” | self-  rated | 15-item, 4-point Likert scale | Korean | undergraduates, South Korea: 0.908 | ^[47]^ |
| eHealth Literacy Scale [eHEALS] | Norman and Skinner ^[43]^ | [1] traditional literacy; [2] computer literacy; [3] information literacy; [4] health literacy; [5] media literacy; [6] science literacy | “...to provide a general estimate of consumer eHealth-related skills” | self-  rated | 8-item,  5-point Likert scale | English | youth population, Canada: 0.88 [18]  lay consumers of online COVID-19-related information, Ghana: 0.87 ^[57]^  African American, Latino, American Indian, Asian and  White older adult patients, USA: 0.73 [58] | ^[15,17,28,57-60,80]^ |
|  |  |  |  |  |  | Spanish | older Hispanics, USA: 0.89 ^[62]^ | ^[17,62]^ |
|  |  |  |  |  |  | Korean | Younger adults, South Korea: 0.88 ^[66, 67]^  Older adults, South Korea: 0.49 ^[52]^ | ^[52,66,67]^ |
|  |  |  |  |  |  | Chinese | School age children, China: 0.88 [69]  Adults, Hong Kong: 0.95 ^[63]^  Older adults, China: 0.97 ^[64]^  Internet users, China: 0.82 | ^[63-65]^ |
|  |  |  |  |  |  | Vietnamese | Healthcare workers, Vietnam: 0.96 ^[68]^  Hospital patients, Vietnam: 0.96 ^[55]^ | ^[68, 55]^ |
| eHealth Literacy Assessment [eHLA] Toolkit | Karnoe et al. ^[36]^ | [1] functional health literacy; [2] self-assessed health literacy; [3] familiarity with health and health care; [4] knowledge of health care; [5] familiarity with technology; [6] technology confidence; [7] incentives for engaging with technology | “...to develop a toolkit with the combination of test and self-assessment elements and a toolkit suitable for screening purposes in projects involving eHealth solutions” | self-  rated and performance-based | self-rated: 86-item, 4-point Likert scale  performance-based: 10-item  performance-based: 10-item, | Danish | adult general Dutch population, Netherlands | ^[36]^ |
| eHealth Literacy Questionnaire [EHLQ] | Kayser et al. ^[33]^ | [1] using technology to process health information; [2] understanding of health concepts and language; [3] ability to actively engage with digital services; [4] feel safe and in control; [5] motivated to engage with digital services; [6] access to digital services that work; [7] digital services that suit individual needs | “…to support researchers, developers, designers, and governments to develop, implement, and evaluate effective digital health interventions” | self-  rated | 35-item, 4-point Likert scale | Danish and English | Adults, Denmark: >0.7 ^[33]^ | ^[21,33]^ |
|  |  |  |  |  |  | Chinese | China: 0.952  ^[69]^ |  |
| electronic Health Literacy Scale [e-HLS] | Seckin et al. ^[12]^ | [1] communication; [2] trust; [3] action | “... for users of digitally provided health information” | self-  rated | 19-item, 5-point Likert scale | English | Older adults, USA: 0.93 ^[12]^ | ^[12]^ |
| Mobile eHealth Literacy Questionnaire | Guo et al. ^[51]^ | [1] eHealth literacy [eHL]; [2] mHealth literacy [mHL]; [3] mobile eHealth preference | “...recognizing the relationship between patient literacy and chronic disease outcomes” | self-  rated | 20-items, 5-point Likert scale | English | Type 2 Diabetes Mellitus patients, Taiwan: 0.927 for eHL, 0.927 for mHL, 0.847 for mobile eHealth preference | ^[51]^ |
| Readiness and Enablement Index for Health Technology [READHY] Tool | Kayser et al. ^[33]^ | 7 eHealth Literacy  Questionnaire [eHLQ] dimensions: [1] using technology to process health information; [2] understanding of health concepts and language; [3] ability to actively engage with digital services; [4] feel safe and in control; [5] motivated to engage with digital services; [6] access to digital services that work; [7] digital services that suit individual needs;  4  Health Education Impact Questionnaire [heiQ] dimensions: [1] self-monitoring and insight; [2] constructive attitudes and approaches; [3] skill and technique acquisition; [4] emotional distress;  2 HLQ dimensions: [1] understanding and support by healthcare providers; [2] social support for health; | “...to assess the readiness of potential  users of health technologies and digital health services, as well  as their degree of enablement” | self-rated | 65-item, 4-point Likert scale | English | - | ^[33]^ |
| Telehealth Literacy Screening Tool [TLST] | Gillie et al. ^[27]^ | [1] biopsychosocial background including access to technology; [2] technological literacy screening; [3] eHealth literacy screening | “...to better understand the unique barriers that older, low telehealth literate and resource-challenged populations face in telehealth utilization” | self-  rated | 16-item, 2-point [0 to 2] Likert scale | English | - |  |
| transactional eHealth literacy instrument | Paige et al. ^[37]^ | [1] Functional eHealth literacy; [2] Communicative eHealth literacy; [3] Critical eHealth literacy; [4] Translational eHealth literacy | “…to measure perceived skills related to the capacity to understand, exchange, evaluate, and apply health information from online multimedia” | self-rated | 18-item, 4-point Likert scale | English | USA: 0.87-0.92 |  |

^a^ previously known as Senior Digital Literacy Evaluation [SDLE]
